# Genetic region characterization (*Gene RECQuest*) - software to assist in identification and selection of candidate genes from genomic regions

**DOI:** 10.1186/1756-0500-2-201

**Published:** 2009-09-30

**Authors:** Rajani S Sadasivam, Gayathri Sundar, Laura K Vaughan, Murat M Tanik, Donna K Arnett

**Affiliations:** 1 Division of Health Informatics and Implementation Science, Quantitative Health Sciences, University of Massachusetts Medical School, USA; 2 Department of Electrical and Computer Engineering, School of Engineering, University of Alabama at Birmingham, Birmingham, Alabama, USA; 3 Section on Statistical Genetics, Department of Biostatistics, School of Public Health, University of Alabama at Birmingham, Birmingham, Alabama, USA; 4 Department of Epidemiology, School of Public Health, University of Alabama at Birmingham, Birmingham, Alabama, USA

## Abstract

**Background:**

The availability of research platforms like the web tools of the National Center for Biotechnology Information (NCBI) has transformed the time-consuming task of identifying candidate genes from genetic studies to an interactive process where data from a variety of sources are obtained to select likely genes for follow-up. This process presents its own set of challenges, as the genetic researcher has to interact with several tools in a time-intensive, manual, and cumbersome manner. We developed a method and implemented an effective software system to address these challenges by multidisciplinary efforts of professional software developers with domain experts. The method presented in this paper, *Gene RECQuest*, simplifies the interaction with existing research platforms through the use of advanced integration technologies.

**Findings:**

*Gene RECQuest *is a web-based application that assists in the identification of candidate genes from linkage and association studies using information from Online Mendelian Inheritance in Man (OMIM) and PubMed. To illustrate the utility of *Gene RECQuest *we used it to identify genes physically located within a linkage region as potential candidate genes for a quantitative trait locus (QTL) for very low density lipoprotein (VLDL) response on chromosome 18.

**Conclusion:**

*Gene RECQuest *provides a tool which enables researchers to easily identify and organize literature supporting their own expertise and make informed decisions. It is important to note that *Gene RECQuest *is a data acquisition and organization software, and not a data analysis method.

## Background

Complex genetic diseases are due to common variants acting alone or in combination with other genes or the environment to cause disease. For the past several years, genetic linkage has been a mainstay for the analysis of these complex diseases. Although there has been some discussion about the future of genetic linkage studies, there is little debate that genetic linkage studies have had tremendous success in identifying regions of the genome that contribute to a wide variety of complex phenotypes [1]. However, identifying the gene (or genes) underlying a linkage peak or in a region of association which drive the association remains a significant challenge.

Linkage studies typically identify regions of association that cover 10-30 cM, which can contain up to 300 genes and be 10-30 Mb in length [2]. In order to identify the causal variant, these regions must then be subjected to fine-mapping, where the area under each region is saturated with additional molecular markers, and these markers are then tested for association with the trait in question. This technique can reduce the area under each region and result in dozens of putative candidate genes for each region. These reduced regions are then subjected to sequence analysis to further refine the search area. Numerous sequence variants, both in coding and non-coding regions, can exist within each region. Because a complex trait region can result from several variants within the same region, each variant must be tested independently for functionality, as well as combinations of all the variants. With a linkage study of a complex trait, multiple regions are expected to be identified, resulting in several hundred, if not thousands, of putative candidate loci, and necessitating fine-mapping and subsequent sequencing for numerous genomic regions, which could be costly and time prohibitive [3-6].

**Figure 1:**
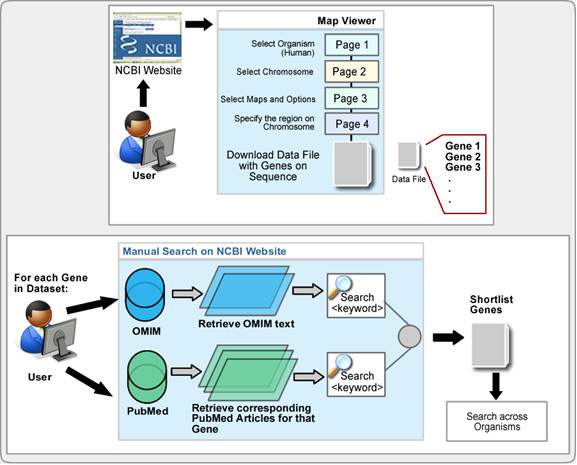
**Workflow of manual linkage studies research activities**.

A commonly used method in selecting genes from a linkage study for follow-up is to identify the genes that lie within the region of interest and select genes from this list for further analysis based on some criteria such as previously demonstrated association with the phenotype. Typically, biological investigators will undertake a manual process such as that depicted in Figure 1, in which researchers browse through multiple pages in the Map Viewer tool of NCBI, selecting the organism, chromosome, maps and other options, and specifying the region of chromosome to download a data file with a list of genes on sequence. For each gene, genetic researchers will then search and retrieve information such as individual PubMed citations and OMIM summaries, which are searched manually for the key words of interest. This process is hampered by the large number of genes in a linkage region, and the difficulty involved in identifying and cataloguing each of those genes.

In an effort to make this gene selection process more efficient, streamlined and organized, we have developed a software system called **GENE**tic **RE**gion **C**haracterization **Quest **(Gene RECQuest). For genes located within a region of interest, *Gene RECQuest *automatically retrieves and stores the PubMed and OMIM citations using XML and Web Services, and allows for a key phrase-based search using database technologies. To demonstrate the utility, we applied our method to a recently identified linkage peak for VLDL response on chromosome 18.

## Implementation

Our overall goal was to design and develop a web-based tool to integrate and customize the existing tools (NCBI web tools) to simplify and streamline genetic linkage studies. We approached the problem with a user-centered design [7] and modified service-oriented architecture approach that is described [8-11]. Full instructions for use are available on the software's website and key implemented features are as follows:

### Integration with Map Viewer

**Figure 2:**
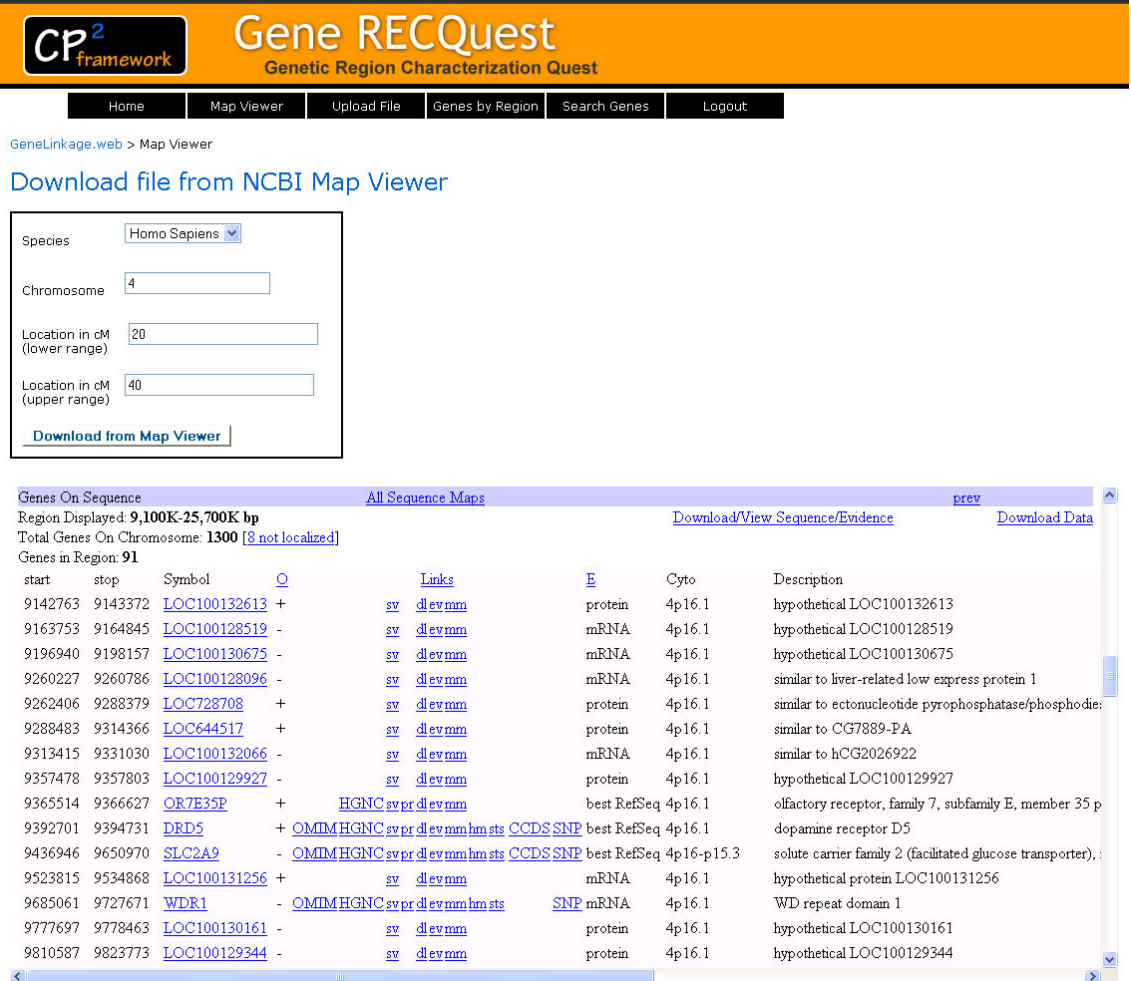
***Gene RECQuest *Map Viewer Interface**.

Integration with the Map Viewer tool in *Gene RECQuest *allows for the reduction of the number of steps required to interact with Map Viewer. Map Viewer is a genome analysis tool provided by the NCBI. Map Viewer can be used to view chromosome maps of many organisms, including humans, and also to identify and localize genes and other biological features [12]. Although NCBI allows the user to view the maps and download the sequence data within a few clicks (without actually typing information), the user has to go through several pages that have more information than the researcher needs for his or her purpose. To reduce the number of steps, we have designed a single interface in which the user will be able to set the parameters for displaying the maps in a single step. The *Gene RECQuest *system (Figure 2) navigates the user to the appropriate Map Viewer from which the user can download the chromosomal data, which will be uploaded to the *Gene RECQuest *MySql database for further analysis. Unfortunately, as the output format of Map Viewer is inconsistent, we were not yet able to integrate the Map Viewer download to the file upload system of *GeneRECQuest*. To overcome this inconsistency, we introduced a step where the user has to format the Map Viewer output before uploading to the *GeneRECQuest *system.

### Integration with NCBI Web Services

NCBI Web Services provide a Web Service Description Language (WSDL)-based Application Programmable Interface (API) to a collection of Entrez E-utilities. The *Gene RECQuest ***NCBI tools-as-services wrapper **uses the NCBI WSDL-API to access and retrieve the necessary information (Gene, OMIM, and PubMed information) from the NCBI database. Due to the amount of data and restrictions set by NCBI, the initial search must be conducted during "off" hours, thus registration with email address is used to notify a user when the search is complete. Integration with NCBI (WSDL)-based API in *Gene RECQuest *allows integrating and automating the access of data from the NBCI databases. For this purpose, we have developed a Windows service as part of *Gene RECQuest *that automatically updates the necessary NCBI data to the MySql database whenever new genes are uploaded to the system. Updating the data to the MySql databases allows us to implement the user-friendly search application using the most recent data available.

### User-friendly search through PubMed and OMIM articles

The implementation of the user-friendly search application has two parts:

#### Setting up a database to collect and organize data

**Figure 3:**
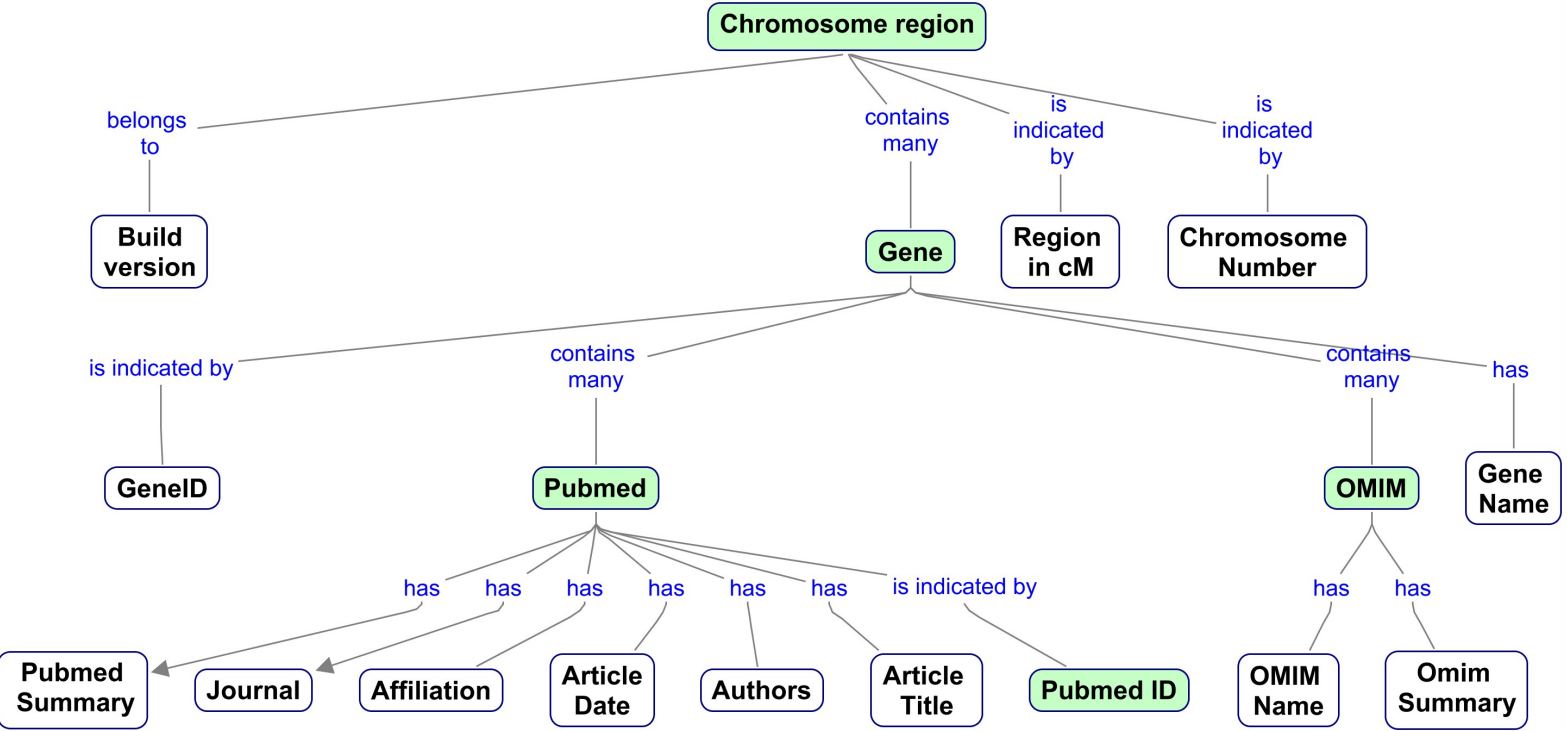
**Concept map of *Gene RECQuest *MySql Database Model for organizing and searching NCBI data**.

The open-source MySql database is used to collect and organize the information accessed from the NCBI web services. For this purpose, we designed a relational data model organizing the NCBI information and implemented the model in MySql. Figure 3 shows a concept map depicting the relationship where the four main entities (Gene, Chromosome region, OMIM, and PubMed) have been highlighted.

#### Key word or key phrases based search interface

One of the key features of this software is the ability to easily and quickly search for key words contained in the literature associated with genes in the chromosomal region of interest. This feature can be found in the "Search Genes" tab on the *Gene RECQuest *website. The user simply selects the region of interest from their list and uses the search form to find genes of interest. We leverage the Boolean full text search feature of MySql database to implement the *Gene RECQuest *full text search on PubMed and OMIM articles for each gene in a region. Instructions are provided to help the researcher construct the search query. Briefly, the researcher can either search using key words or phrases of interest, but must specify variants of interest (e.g. searching for singular and plural variations). The search results first provide a listing of genes that contained both OMIM annotations and PubMed articles matching the search phrases in the same format as the "Genes in Regions" list.

**Figure 4:**
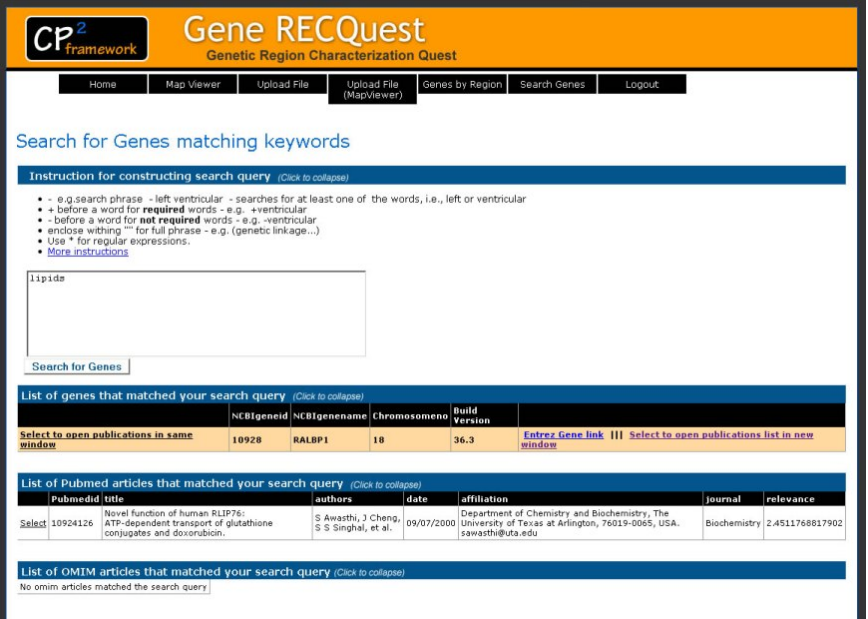
***Gene RECQuest *User-Friendly Search Interface**.

The researcher can easily navigate the search results by clicking on each gene to see the OMIM and PubMed articles that contain the search phrases. The PubMed articles information includes the PubMedID, title, authors, date of publication, journal of publication, and the authors' primary affiliation. The researcher can see the abstract by clicking on each article. A link to the PubMed web page for each article is also provided. The OMIM information includes the OMIMID (MIM number) and the OMIM name. Researchers can also see the OMIM summary by clicking on each OMIM and a link to the OMIM page is also provided. Figure 4 shows an example of the search results web page.

### Application to a research question

We chose to use one of our own research questions to illustrate the utility of *Gene RECQuest*. As a part of the Genetics of Lipid reducing drugs and Diet Network (GOLDN) project, a QTL analysis was conducted for VLDL response to a high fat meal (see [13] for full details). The study was conducted in 1254 individuals. Briefly, the high fat challenge meal was determined by body surface area, and contained 700 kilocalories per m^2 ^of body surface area. The meal composition was 83% of calories from fat, 14% from carbohydrates, and 3% from protein. VLDL was measured twice (the day before and the day of the meal), and at 3.5 and 6 hours after the meal using proton nuclear magnetic resonance (NMR) spectroscopy. The VLDL response was calculated using a mixed model where a growth curve was created across the four measurement points. Variance components linkage analysis was implemented using the program SOLAR (Sequential Oligogenic Linkage Analysis Routines) for the VLDL response trait.

**Figure 5:**
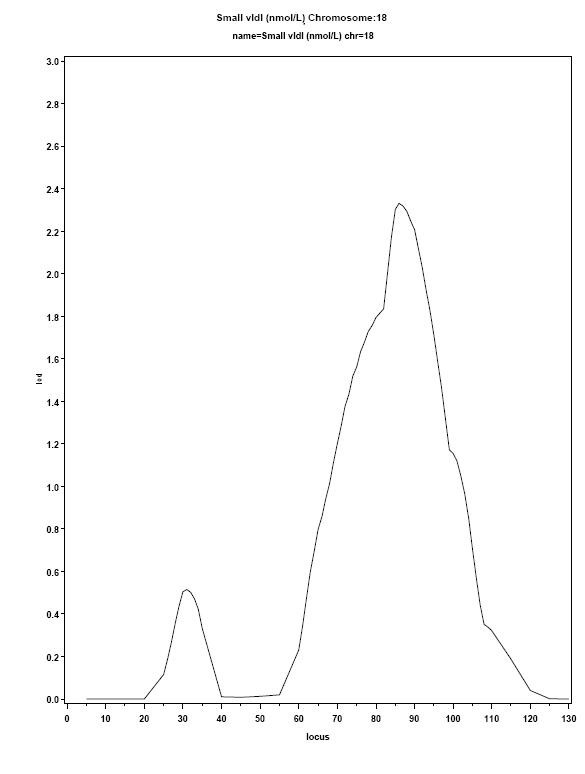
**Chromosome 18 LOD plot for VLDL response to high fat milk shake (GOLDN study)**. Although this QTL peak would not be considered significant (LOD >3), it is suggestive. The region spanning from 60-q20 cM was included in this analysis.

The most promising region identified in this study was located on chromosome 18, with a maximum LOD score of 2.4 (Figure 5). For illustrative purposes, we chose to include the entire region under the QTL peak, from 60 to 120 cM. This large, 60 cM region contains over 230 genes (480 total genes on chromosome 18), many of which may be likely candidates. The task of manually identifying each gene, researching associated publications, and tracking known associations with human diseases through OMIM is monumental. Of the 230+ annotations in the region, there are136 genes, 70 hypothetical, 16 open reading frames (ORFs), 10 pseudo genes, 3 micro RNAs and 2 anticodons. The list of genes retained by *Gene RECQuest *in the "Genes by Region" tab contained 89 genes and 1 micro RNA, each with a link on the left to "select" the related PubMed and OMIM citations, and a link on the right that opens the Entrez Gene annotation in a new window.

### Search for potential candidate genes

Key word searches for terms associated with the phenotype of interest were quickly and easily conducted, and a list of genes was generated (Table 1). Search terms included lipid(s), lipoprotein(s), triglyceride(s), blood pressure and postural hypertension. Several of the genes (indicated by bold in Table 1) were identified through multiple key word searches. Of particular interest are the genes that were contained in the annotations for 4 out of the 5 key words, lipase (LIPG), melanocortin 4 receptor (MC4R) and SMAD family member 2 (SMAD2), all of which have strong support for possible involvement in lipid metabolism.

**Table 1:** Gene RECQuest results for key word search.

**Chromosome 18 region 60-120 cM total 237 genes in region & 480 on chromosome 18**				
**Lipoprotein(s)**	**Triglyceride(s)**	**Lipid(s)**	**"Blood Pressure"**	**Hypertension**
DCC	**LIPG**	ACAA2	SLC14A2	**LIPG**
**LIPG**	LMAN1	ATP8B1	NEDD4L	LMAN1
**MC4R**	**MC4R**	BCL2	**MC4R**	**MC4R**
***SERPINB2***		CD226	***SERPINB7***	MEX3C
***SERPINB5***		CYB5A		MRPS5P4
***SERPINB3***		DCC		NEDD4L
**SMAD2**		ELP2		***SERPINB7***
SMAD4		FVT1		**SMAD2**
		KRT8P5		SOCS6
		**LIPG**		
		MALT1		
		**MC4R**		
		MBP		
		MRPS5P4		
		NEDD4L		
		PIGN		
		PIK3C3		
		POL1		
		***SERPINB2***		
		SLC39A6		
		**SMAD2**		
		SMAD4		
		STARD6		
		SYT4		
		VPS4B		

Bold indicates genes that are present for more than 4 of the terms.

## Conclusion

It is important to note that *Gene RECQuest *is a data acquisition and organization software, and not a data analysis method. There is a multitude of data analysis methods designed to aid in the selection of candidate genes from lists generated by linkage or association studies [14-17]. Many of these methods rely on some sort of annotation analysis, either comparison to a list of known genes (e.g. ENDEAVOUR) [18] or over-representation enrichment (e.g. GSEA) [19], to identify genes of interest. Often methods that utilize the same input data and annotation sources produce very different output. As a result, it has been suggested that the best approach is to use multiple prioritization tools [20]. Although these methods are potentially very powerful and have a place in one's toolbox, investigators often approach them with hesitation and want to read relevant literature to aid in their decisions. *Gene RECQuest *provides a tool which enables researchers to identify and organize literature supporting their own expertise easily, and make informed decisions.

## Availability and requirements

• Project name: *Gene RECQuest*

• Project home page: 

• Anonymous accounts (no e-mail address for registration is needed): 

• Operating systems: any OS (that has an internet browser application)

• Programming language: .Net, ASP.Net, C#, MySQL

## Abbreviations

API: Application Programmable Interface; GOLDN: Genetics of Lipid lowering drugs and Diet Network; NCBI: National Center for Biotechnology Information; NMR: Nuclear magnetic resonance; OMIM: Online Mendelian Inheritance in Man; QTL: Quantitative trait locus; SOLAR: Sequential Oligogenic Linkage Analysis Routines; VLDL: Very low density lipoprotein cholesterol; WSDL: Web Service Description Language; XML: Extensible Markup language.

## Competing interests

The authors declare that they have no competing interests.

## Authors' contributions

RS and LKV prepared the manuscript. RS designed and developed the *Gene RECQuest *system. GS prepared the throw-away prototypes and assisted in the task analysis of the genetic linkage studies. MMT oversaw the software engineering of the *Gene RECQuest *system. LKV and DA provided domain expertise for the development of the system. DA served as the project lead. All authors read and approved the manuscript.
